# Preliminary results from a crowdsourcing experiment in immunohistochemistry

**DOI:** 10.1186/1746-1596-9-S1-S6

**Published:** 2014-12-19

**Authors:** Vincenzo Della Mea, Eddy Maddalena, Stefano Mizzaro, Piernicola Machin, Carlo A Beltrami

**Affiliations:** 1 Department of Mathematics and Computer Science, University of Udine, Italy; 2 ULSS 7 Pieve di Soligo, Italy; 3 Department of Medical and Biological Sciences, University of Udine, Italy

## Abstract

**Background:**

Crowdsourcing, i.e., the outsourcing of tasks typically performed by a few experts to a large crowd as an open call, has been shown to be reasonably effective in many cases, like Wikipedia, the Chess match of Kasparov against the world in 1999, and several others. The aim of the present paper is to describe the setup of an experimentation of crowdsourcing techniques applied to the quantification of immunohistochemistry.

**Methods:**

Fourteen Images from MIB1-stained breast specimens were first manually counted by a pathologist, then submitted to a crowdsourcing platform through a specifically developed application. 10 positivity evaluations for each image have been collected and summarized using their median. The positivity values have been then compared to the gold standard provided by the pathologist by means of Spearman correlation.

**Results:**

Contributors were in total 28, and evaluated 4.64 images each on average. Spearman correlation between gold and crowdsourced positivity percentages is 0.946 (p < 0.001).

**Conclusions:**

Aim of the experiment was to understand how to use crowdsourcing for an image analysis task that is currently time-consuming when done by human experts. Crowdsourced work can be used in various ways, in particular statistically agregating data to reduce identification errors. However, in this preliminary experimentation we just considered the most basic indicator, that is the median positivity percentage, which provided overall good results. This method might be more aimed to research than routine: when a large number of images are in need of ad-hoc evaluation, crowdsourcing may represent a quick answer to the need.

## Background

Crowdsourcing, i.e., the outsourcing of tasks typically performed by a few experts to a large crowd as an open call, has been shown to be reasonably effective in many cases, like Wikipedia, the Chess match of Kasparov against the world in 1999, and several others. Several crowdsourcing platforms (Amazon Mechanical Turk being probably the most known) have also appeared on the Web: they allow requesters to post the tasks they want to crowdsource and workers to perform those tasks for a small reward.

One classical crowdsourcing topic is image recognition, in the form of image tagging and moderation for usage in image databases, forums, etc. However, crowdsourcing has been also very recently applied to biomedical image analysis in fields like retinal fundus photography classification, malaria parasite quantification, CT colonography [1-3].

The aim of the present paper is to describe the setup of an experimentation of crowdsourcing techniques applied to the quantification of immunohistochemistry on breast samples, and its preliminary results.

## Methods

### Images

Fourteen images were acquired from breast cancer specimens stained with MIB1, using an Olympus Provis AX70 microscope at 40× and a Leica DFC320 camera, set up for acquiring images 1044 × 772 pixels. MIB1 was chosen just as example of immunohistochemical marker.

For obtaining the gold standard, positive, negative and other nuclei were manually identified on each image by a pathologist using the image analysis software ImageJ. For this task, a macro has been developed to support the pathologist in clicking on nuclei, marking the clicked points on the image, and recording coordinates and type of the nuclei on a text file for further processing.

One of the 14 images was used in the preliminary evaluation of the developed crowdsourcing applications, while the other 13 were used for the real experimentation.

### Crowdsourcing application

While Amazon Mechanical Turk [4] is perhaps the largest crowdourcing platform on the market, we were not able to use it for the experimentation because it only accepts task offers from USA citizens. For this reason we selected another well known platform, Crowdflower [5], which has been already used for analysing tuberculosis cells as well as neurons (unpublished results)[6].

Crowdflower acts as a broker between requesters (i.e., who builds a crowdsourcing job and orders the task) and contributors (i.e., members of the crowd that work on tasks). In its intermediate role, Crowdflower provides a platform to develop web-based task interfaces, manages contributors and quality, and assembles results. At the core of the platform there is a language for specifying the web interface, CML (Crowdflower Markup Language) that we used, together with HTML, CSS and Javascript, to develop our own application.

The application for immunohistochemistry count is very simple: after a small instructions section, it shows an image and allows for clicking on positive and negative nuclei. Since the image is larger than most screens, to make the task more adequate, every image has been rotated to be vertical and inserted in a scrollable canvas. When finished, the contributor ends the task and control passes back to the crowdsourcing platform.

**Figure 1:**
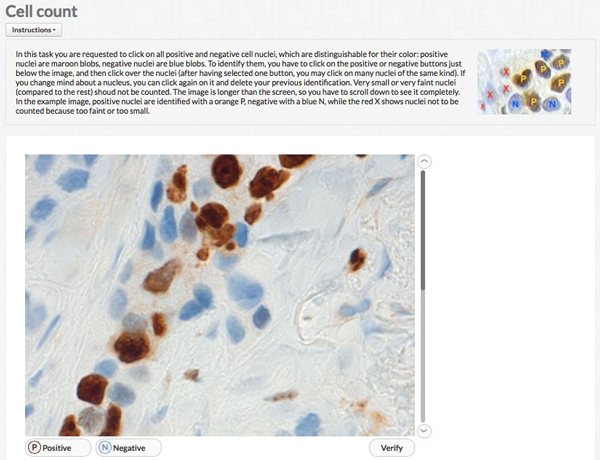
**Crowdsourcing application interface**. The screenshot depicts the interface before any nucleus selection.

**Figure 2:**
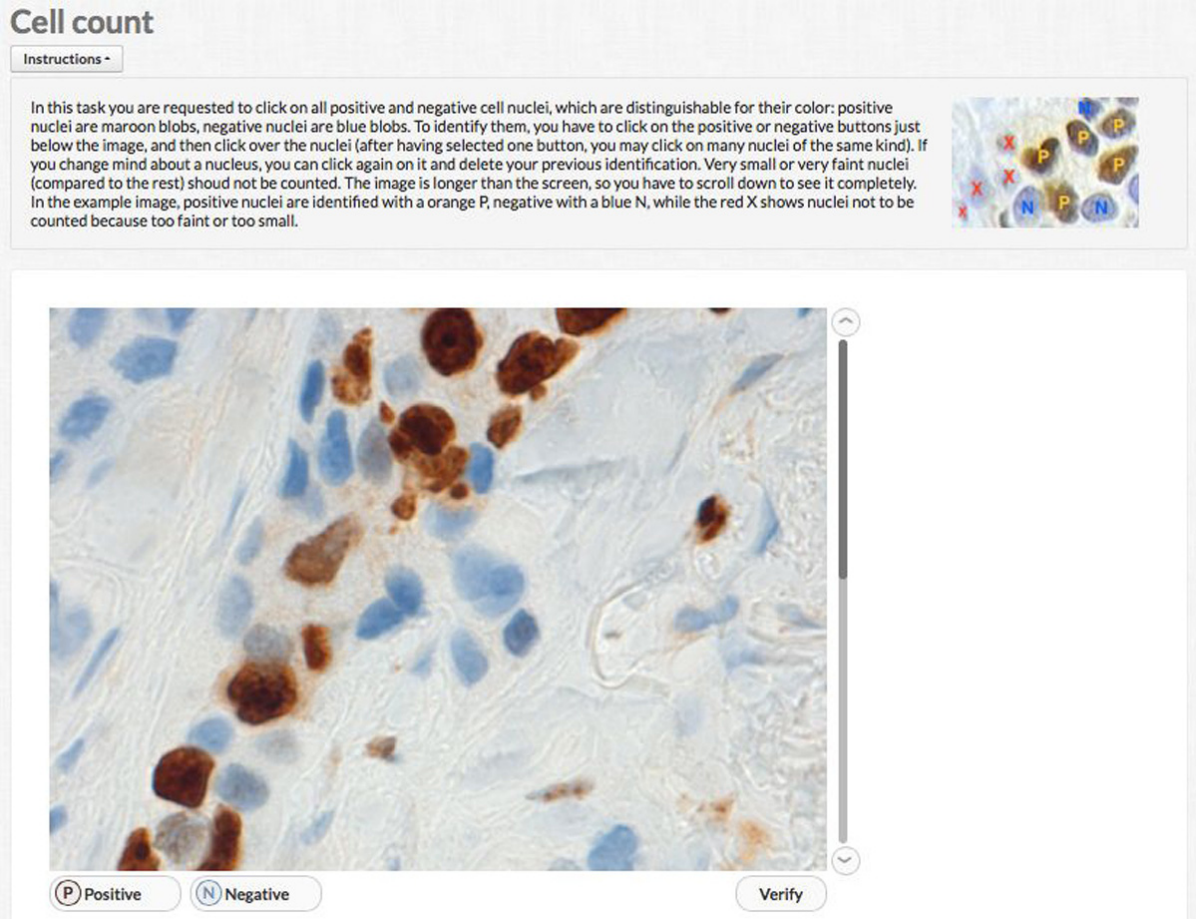
**Crowdsourcing application interface during input**. The screenshot depicts the interface after some nucleus selection.

Figure 1 shows the interface before starting any count, Figure 2 after having identified some cells.

Since one of the issues with crowdsourcing is the quality of the executed task, a common practice is to insert some quality indicator in the task, e.g., questions that the contributor is expected to answer only in one way. The selected platform has a specific approach to manage those questions, called "gold", based on which the contributor is paid or not, and his/her "trust score" is increased or decreased, to qualify him/her for further tasks.

In our case, it is difficult to have such kind of quality indicator. However, we implemented two controls to be sure that contributors at least attempted to do the right thing: a minimum number of cells (sum of positive and negative) had to be counted, and at least one vertical coordinate had to be in the lower part of the image. Without any control, the contributor that eventually just started and ended the task without clicking in any place would have been paid anyway.

### Statistical analysis

The experiment included one pilot to check whether the application was correctly functioning and to identify possible issues not covered in the instructions for contributors. Each run was made on a single image, not reused for further runs.

The main experiment involved 13 images, each one representing a task unit. The platform was instructed to collect 10 "judgments" (i.e., task results) for each image, letting workers to execute up to 13 units, but not more than one time each.

For each judgment we collected the country of workers, time spent for each unit, the number of times they tried ending the task before achieving the quality indicator values needed to pass the quality test, the number of afterthoughts (i.e., clicking again on an already selected nucleus to delete selection), and the coordinates of every selected positive and negative nucleus.

For each image and contributor we calculated the number of positive and negative nuclei and the positivity percentage (defined as ratio between number of positive nuclei and total number of nuclei), and compared it with the gold standard obtained by manual count.

Finally, we summarised results for each image, by calculating the median value for positivity percentage. This has been taken as main outcome of the experiment, according to the principle that the median is the best reflection of the crowd's estimate [7].

The Spearman correlation between gold standard and median positivity on the 13 images set was also calculated, as well as linear regression.

## Results

After the two test runs, the main experiment has been launched with all images at the same time, thus offering 13 task units to the contributor crowd. The total number of 130 judgments has been obtained after 58 minutes from launch.

Contributors were in total 28, from 18 different countries; each contributor evaluated 4.64 images on average, and spent 2:43 minutes for each on average.

**Figure 3:**
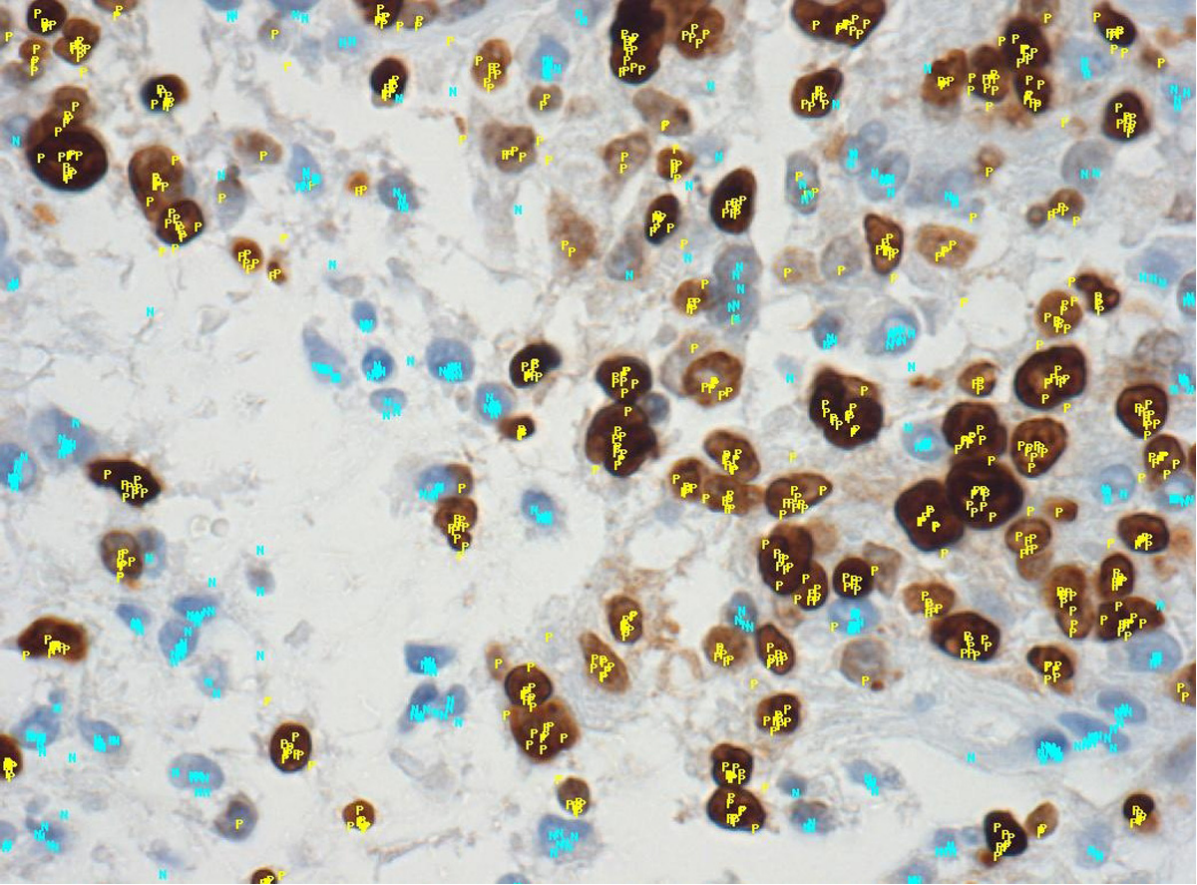
**A sample image with crowd's nuclei selections**. Light blue "N" are negative nuclei according to the crowd, yellow "P" are positive.

Figure 3 shows a sample image with crowd evaluations plotted on it.

**Figure 4:**
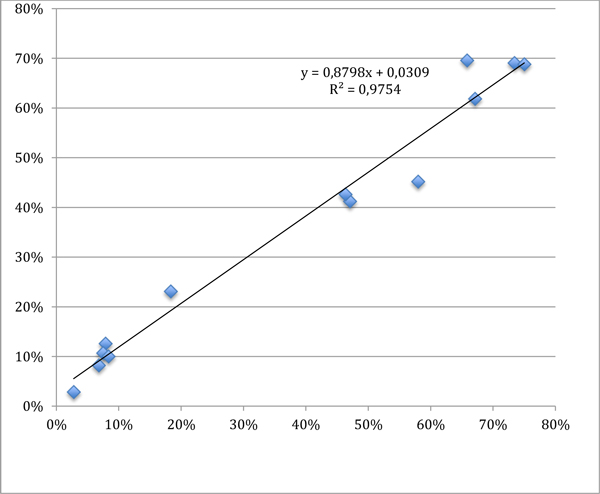
**Relationship between gold standard (X) and crowdsourced evaluations (Y)**.

Table 1 shows the comparison of gold positivity percentages vs. crowdsourced median percentages, which are also plotted in Figure 4. Spearman correlation is 0.946 (p < 0.001); however, the sample is small and thus this high value should be taken with caution.

**Table 1:** positivity in each image.

Image	Gold standard	Crowdsourced
1	65.83%	69.62%

2	73.43%	69.10%

3	47.06%	41.26%

4	18.33%	23.14%

5	75.00%	68.83%

6	67.11%	61.90%

7	7.88%	12.63%

8	6.78%	8.25%

9	7.48%	10.71%

10	8.33%	10.05%

11	2.78%	2.88%

12	46.34%	42.66%

13	57.97%	45.23%

Contributors rarely tried to end the task before reaching the minimum number of cells, and also rarely changed mind after having selected a nucleus.

## Conclusions

Aim of the experiment was to understand how to use crowdsourcing for an image analysis task that is currently time-consuming when done by human experts, and somewhat difficult, although feasible, if done by software. Crowdsourced work can be used in various ways, in particular relying on the crowd to reduce identification errors (e.g., by considering the most selected cells, or the cells selected above a threshold, etc). However, in this preliminary experimentation we just considered the most basic indicator, that is the median positivity percentage, which provided overall good results. There is however a trend to overestimate negative nuclei (about 3% according to linear regression), which was somewhat expected because of the presence of stromal cells and lymphocytes that must not to be counted. Further work will be carried out to investigate the details of this overestimation, although other reports exist regarding the same issue when comparing manual vs. automated evaluation [8], since recognising cells that should not be counted is an issue for both an untrained human and a software. Anyway, if adopting the positivity cutoff at 15% as defined in [9], no false positives or false negatives have been identified in our small sample.

While we did not have access to full information on the crowd, in the report provided by the platform we found that people participating in the experiment came from 18 different countries. Unfortunately, no information is released about education of the workers. Although not sufficiently detailed, this data allows at least to suppose they were heterogeneous in knowledge, and this goes towards one of the principles behind the so-called "wisdom of the crowd", i.e., heterogeneity [7].

Since the task of quantifying immunohistochemistry is time-consuming and prone to errors, with constrasting reports when compared to automated image analysis [8,10,11], the presented approach tries to propose an unorthodox way to quantification: instead of relying on expert and expensive personnel like pathologists, evaluation could be made by a less expensive, untrained crowd working on digital images and statistically aggregated.

One point of discussion is the fact of using anonymous workers of unknown qualification on a medical task that can be quite sensible.We adopt a pragmatic attitude towards this issue: we set up to understand if and how crowdsourcing can be used to work as effectively as experts. Or perhaps even more effectively, as it has been shown in other domains [12]. If the results will prove that the approach is feasible, the discussion on the opportunity of such an approach will follow, and will be based on solid data.

However, the proposal might be more aimed to research than routine: when a large number of images are in need of ad-hoc evaluation, providing that they do not involve too specific knowledge, crowdsourcing may represent a quick answer to the need.

## Competing interests

The authors declare that they have no competing interests.

## Authors' contributions

VDM conceived the experiment, analysed data and drafted the paper. EM and SM designed the protocol; EM also implemented the application and contributed to data analysis. PM provided the gold standard by annotating the images. CAB provided support in discussing the anatomo-pathological issues related to the experiment. All authors reviewed and commented the paper.
